# A cluster-randomized trial of a brief multi-component intervention to improve tobacco outcomes in substance use treatment

**DOI:** 10.1186/s13011-023-00539-w

**Published:** 2023-06-16

**Authors:** Joseph Guydish, Caravella McCuistian, Sindhushree Hosakote, Thao Le, Carmen L. Masson, Barbara K. Campbell, Kevin Delucchi

**Affiliations:** 1 Philip R. Lee Institute for Health Policy Studies, University of California San Francisco, 490 Illinois St., Floor 7, San Francisco, CA 94158 USA; 2 Department of Psychiatry and Behavioral Sciences, University of California San Francisco, San Francisco, USA; 3 Division of General Internal Medicine & Geriatrics, Oregon Health and Sciences University, Portland, USA

**Keywords:** Tobacco control, Smoking cessation, Substance use treatment, Policy

## Abstract

**Background:**

Smoking prevalence is high among people in substance use disorder (SUD) treatment, and program interventions to address smoking are often complex and lengthy. This cluster-randomized trial tested whether a brief multi-component intervention impacted tobacco outcomes among staff and clients.

**Methods:**

Seven SUD treatment programs were randomly assigned to the multi-component intervention or to waitlist control. The 6-month intervention included a leadership motivation assessment, program incentives, 4 staff training sessions and a leadership learning community session. Survey data were collected from staff and clients at pre- and post-intervention. Outcomes were first compared across condition (intervention vs waitlist control), and then examined pre- to post-intervention with condition collapsed.

**Results:**

Staff in the intervention (*n* = 48) and control conditions (*n* = 26) did not differ at post-intervention on smoking prevalence, self-efficacy to help clients quit, or practices used to help clients quit smoking. Intervention clients (*n* = 113) did not differ from controls (*n* = 61) in smoking prevalence or receipt of tobacco services. Pre-post comparisons collapsed across condition showed a decrease in client and staff smoking prevalence, which could not be attributed to the intervention, and a decrease in client receipt of cessation medication.

**Conclusion:**

The brief multi-component intervention did not support changes in smoking prevalence or in tobacco-related services received by clients. Other intervention features are needed to reduce smoking among SUD clients.

**Trial registration:**

Randomization occurred at the program level and outcomes measured are program-level measures. Accordingly, the trial is not registered.

**Supplementary Information:**

The online version contains supplementary material available at 10.1186/s13011-023-00539-w.

## Introduction

The prevalence of combustible cigarette smoking among adults in the United States (US) is 12.5% [1]. Among persons with serious psychological distress, smoking prevalence is 35.2% [2]. Among persons with substance use disorders (SUDs), smoking prevalence differs depending on which SUDs are included. Using 2014 National Survey on Drug Use and Health (NSDUH) data, Weinberger et al. [3] estimated a 55.48% smoking rate among persons meeting criteria for any illicit SUD, excluding alcohol use disorder (AUD). Han et al. [4] using NSDUH data from 2019, and including both cannabis use disorder (CUD) and AUD in the sample, estimated a 35.8% smoking prevalence.

The Substance Abuse and Mental Health Services Administration (SAMHSA, 2021) [5] estimates that 10% of those who need SUD treatment receive such treatment, so that persons entering SUD treatment represent a small subset of all those who meet SUD diagnostic criteria. A survey of 1,700 clients in 24 SUD treatment programs in 14 states reported a 77.6% smoking prevalence [6]. A recent survey of clients enrolled in 20 California residential SUD treatment programs found that 68.9% were current smokers [7]. These estimates reflect a gradient in smoking rates from 14% in the general population, to 35–55% among persons with mental health or substance use problems, to about 70% among those in SUD treatment.

Differences in smoking rates reflect health disparities [8] and social justice issues [9], and suggest that decades of tobacco control efforts have had limited impact in this group. High smoking rates also concern SUD treatment payors and providers, as the health and economic costs of smoking are concentrated in this population. Smokers with SUDs smoke more heavily than other smokers [10], have a harder time quitting smoking [11] even while attempting to quit at rates similar to the general population [12, 13], are more likely to relapse to drug use (Weinberger et al. 2015; [14]), and more often die of tobacco-related causes [15].

Several states have implemented tobacco free policies to address smoking in SUD treatment. New Jersey mandated tobacco free grounds in residential SUD programs in 2001 [16], New York implemented tobacco free grounds in all state licensed SUD programs in 2008 [17], later followed by both Oregon and Utah [18, 19]. Other initiatives have sought to reduce smoking in SUD treatment without statewide mandates. Texas implemented a community-academic partnership including staff training and access to nicotine replacement therapy (NRT) to encourage tobacco free grounds in behavioral health settings [20, 21]. The California State Tobacco Free Recovery initiative was designed to help residential SUD programs implement tobacco-free policies [22].

These interventions are often complex and time-consuming. They are also likely to be costly, however there are no published reports estimating costs of tobacco-free policy interventions. The New York State policy, with support of the state regulatory authority for SUD treatment, followed a multi-year planning process and provided staff training and NRT to programs [23]. The California initiative engaged programs in an 18-month intervention inlcuding program contracts with specific deliverables, ongoing external consultation, and participation in learning communities [22]. Other studies have used a 6-month intervention, including multiple levels of staff training, consultation, and increased availability of NRT ([24, 25]).

Apart from a statewide mandate, like that in New York, little is known about how to bring these interventions to scale in SUD treatment systems. The California Department of Healthcare Services (CalHHS, 2023) [26] licenses nearly 2,000 SUD treatment programs. The California Tobacco Free Recovery initiative [22] worked with 18 programs during a 4-year period. Applying this intensive intervention to all California SUD programs, at the same rate, would take decades. Shorter, simpler, and low-cost approaches are needed to address smoking in SUD treatment.

While randomized trials have been used to test patient-level smoking cessation interventions in SUD treatment [27], research on broader tobacco policy and training initiatives have relied on document review [19], secondary analyses [16], reports of program directors [17] and staff [28], and pre-post [20] or other observational designs [29]. Similarly, while counseling and medication interventions for individual smokers are evidence-based [30], studies about implementing these strategies in SUD treatment are few [29]. Last, although client smoking rates may seem an obvious outcome measure, few studies of tobacco policy or training initiatives have measured impact on client smoking [22, 23].

The current study recruited residential SUD programs into a cluster-randomized trial of a brief multi-component tobacco intervention. The objective was to assess whether the intervention, modified to increase scalability, may affect staff or client smoking rates, or tobacco-related services received by clients.

## Methods

### Intervention description

The multi-component intervention was based on the Addressing Tobacco Through Organizational Change (ATTOC) model, which targets barriers at multiple levels and supports program leaders through an organizational change process [24, 31]. The approach focuses on leadership, champions, staff training, and consultation. We fielded a simplified intervention focused on leadership motivation, program incentives, staff training, and a leadership learning community.

Leadership commitment to address smoking is a common feature in program-level tobacco interventions (e.g., [32, 33]). During a phone survey of California residential SUD programs concerning tobacco policies, 33 programs expressed interest to address tobacco use [34]. These programs were contacted by email about the study and 10 responded. In phone discussions with each program director, the director was asked about their interest to address smoking among clients, and the study was explained as an intervention to help programs better address smoking in their program. Study procedures, including the number and content of the webinars, survey data collection procedures, and program level incentives were reviewed. Of the 10 program directors, 6 agreed to participate. Two directors asked if other programs in the same agency could participate, bringing the total assessed for eligibility to 12. Four programs declined, one did not complete baseline data collection, and seven programs were randomized (Supplemental Figure 1). The use of the brief phone survey, with following email and phone contacts, signaled that leadership was interested to address smoking in their program.

Each program received a $15,000 incentive, with no restriction on use. Program incentives were disbursed on the same schedule for all programs, so that each program received $5,000 after baseline, $5,000 after 6 months, and the final $5,000 after the 12-month data collection. Financial disincentives create a barrier to organizational change [35], for example when programs are asked to provide additional services without additional reimbursement. The California Tobacco-Free Recovery initiative provided each program a $36,000 incentive, for use without restriction as long as they met contractual requirements [22]. An earlier study provided $11,000 per program to purchase NRT [24]. The New York State tobacco free policy intervention did not incentivize individual programs. However, the state Department of Public Health allocated $4 million for program-level training and technical assistance, and $4 million to supply free NRT for patients without insurance coverage [17, 23].

Each program participated in a sequence of four, one-hour, staff training sessions scheduled approximately once per month. A fifth session, held after the training sequence and near the end of the intervention period, was used as a learning community for program leaderships. Lack of training is commonly cited by staff as a barrier to providing tobacco services to clients ([29, 36–38]), and staff training has been associated with increased provision of tobacco-related services [39, 40].

In the current study, the first training session included the prevalence of smoking in SUD treatment and associated mortality, and summarized data drawn from baseline surveys collected in that program. The first training session was conducted in-person for intervention programs but, due to COVID restrictions, was conducted by webinar for waitlist programs. Following the first training session, program staff were invited by email to 3 additional webinar trainings. In four programs all staff were invited to all trainings, while in 3 programs the Director selected staff to attend. The second training discussed how to talk with clients about their tobacco use, use of the 5As (Ask, Advise, Assess, Assist, and Arrange; AHRQ, [41]), and introduced a toolkit for cessation counseling [42]. The third training reviewed pharmacological treatments and best practices in the use of cessation medications. The fourth training discussed steps for implementing comprehensive tobacco-free policies [43].

Learning communities, with roots in educational systems, are used to promote capacity building and sustainability [44], and were used in the California Tobacco-Free Recovery intervention [22]. The final webinar, occurring after the 4 training webinars, was a virtual learning community attended by program leadership. The one-hour Learning Community session was attended by two research team members (JG, SH) and the director of each program receiving the intervention in that cycle. Discussion was guided by three broad questions: what changes, if any, were made in the program during the intervention period,what factors may further support programs in addressing smoking among clients; and whether the program received guidance or support concerning smoking from state or county SUD authorities. At the end of the webinar the research team reviewed plans for the next, post-intervention, survey data collection.

### Study design

Following baseline, participating programs were randomly assigned to intervention (4 programs) or control conditions (3 programs). Randomization was stratified so that programs from the same agency were not assigned to the same condition. Survey measurement in all programs was at baseline, and at 6- and 12-months post. Baseline data collection occurred from June to August 2019, and final (12 month) data collection occurred from December 2020 to January 2021. The original plan was for data collection at 6-month intervals, however adjustments necessitated by the COVID pandemic extended the interval to about 8 months.

**Fig. 1:**
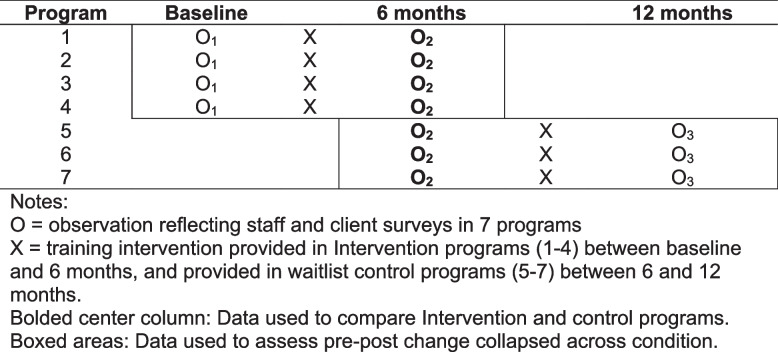
Study design showing clinical trial comparison and pre-post comparison

Intervention programs received the intervention between baseline and 6 months, while waitlist programs received training between 6 and 12 months (see Fig. 1). For the randomized trial, we compared outcome measures between the intervention and control programs using 6-month data (bolded column in Fig. 1). To compare outcomes from pre- to post-intervention, collapsed across condition, we used the data in the boxed areas of Fig. 1.

### Participants

Eligible staff were all full or part-time paid program staff. Eligible clients were those enrolled in the program at the time of data collection. Program directors provided a list of program staff, and the number of program clients at each timepoint, for use in determining response rates.

### Measures

#### Intervention exposure

The research team recorded the names of staff present in each training session and the learning community session. These were used to report number of attendees per session, number of unique staff who participated in any session, and proportion of eligible staff exposed to at least one session.

#### Staff measures

The staff survey asked about demographic characteristics and smoking status (current, former, never). Current smokers reported number of cigarettes per day (CPD) and, as a measure of readiness to quit smoking, whether they intended to quit in the next 30 days, the next 6 months, or were not thinking of quitting [45].

Staff completed the Smoking Knowledge, Attitudes and Practices (S-KAP) survey, which includes scales reflecting staff beliefs about addressing tobacco in the treatment setting (7 items, α  = 0.74), self-efficacy to help clients quit smoking (9 items, α  = 0.72), and practices (8 items, α  = 0.91) used to help clients quit [46]. Individual scale items and response codes are found at https://tinyurl.com/SKAPscale. Responses to each item are scored from 1 to 5, with the mean of items comprising the scale score. Higher scores reflect more positive beliefs about treating smoking, greater self-efficacy to treat smoking, and greater use of practices to treat smoking. All staff completed the Belief scale, while clinical staff completed the Self-Efficacy and Practice scales. Clinical staff included those having an active client caseload, and/or conducting group or individual counseling sessions.

#### Client measures

In addition to demographic characteristics and smoking status, current smokers reported CPD and readiness to quit smoking. To assess tobacco-related services received, clients reported whether any staff member had asked if they smoke. Current smokers and former smokers who quit while in treatment reported whether they had attended a smoking cessation support group (yes/no), and how often their counselor encouraged them to quit smoking or arranged an appointment to discuss quitting (Never vs. Occasionally/Often/Very Often/Always). Clients who received one or more of these three services were coded as having received tobacco-related counseling. Last, smokers and former smokers who quit while in treatment were asked if they received NRT, or other cessation medication, in the program. Answering “yes” to either question was coded as having received cessation medication.

### Procedures

#### Staff surveys

Staff received an initial email invitation, followed by three weekly reminders, to complete the survey. Qualtrics recorded which staff had completed the survey, so that surveys were linked across time for the same person. The research team then talked with each director to increase staff response by, for example, resending emails to non-responders, or having the director discuss the survey in staff meetings. Respondents received a $25 gift card.

#### Client surveys

At baseline, anonymous client surveys were collected during site-visits to each program. Meeting with clients in small groups, research staff reviewed a study information sheet and gave each client a computer tablet with a pre-populated ID number. Clients consented or declined participation using the tablet, completed the 30-min survey, and received a $20 gift card. The California COVID-19 shelter in place order (March 19, 2020) prohibited research staff from visiting programs to administer surveys on-site. Consequently, post-intervention client surveys were completed using mailed paper surveys in 2 of the 4 intervention programs, and in all 3 control programs. Study procedures were approved by the Institutional Review Board of the University of California San Francisco.

### Data analyses

We report demographic and tobacco use characteristics for staff (*n* = 74) and clients (*n* = 174) collapsed across condition at 6 months. Staff outcomes included smoking prevalence, and the mean SKAP Belief, Self-Efficacy, and Practice scale scores. Client outcomes included smoking prevalence, the proportion of clients that had been asked about their smoking status, and the proportions of current smokers (and former smokers who quit while in treatment) that had received tobacco-related counseling or medication in the program.

Randomized trial guidelines are that participant characteristics in each condition should not be compared at baseline [47]. Accordingly, we compared participants in each condition on selected outcomes at 6 months only. This timepoint reflects post-intervention for the intervention group and the end of the waiting period for the control group. Proportions were compared using Pearson’s chi-square tests, and continuous measures were compared using t-tests.

As all 7 programs received the intervention at some point, we also collapsed across condition and compared outcomes pre-post intervention. There were 8 selected outcomes (4 staff outcomes and 4 client outcomes). For each outcome, we used a linear regression model including time (pre-post intervention) as a predictor, adjusted for demographic characteristics (age, gender, race/ethnicity, education) and for nesting of participants within clinic. Generalized estimating equations with logit link were used for dichotomous outcomes, and mixed-effects regression models were used for continuous outcomes. Client analyses assume independent samples because California Medi-Cal pays for 90 days of treatment, so the same client would not have been in treatment at both timepoints. However, the models allowed for correlations within staff who completed surveys at both data collection points. Last, in a sensitivity analysis, the regression models were repeated but removing data for one control program (here identified as Program 5) that implemented tobacco-free policies shortly after baseline.

## Results

### Intervention exposure

Program directors from all 7 programs completed the leadership commitment assessment, and all 7 programs received the program-level incentive.

For the 4 intervention programs, and across all 5 sessions, there were 57 attendees (average 14 per program). These attendees represented 36 unique persons (average 7 per program). Considering all eligible staff, 63.2% (36/57) attended at least 1 training. The range of unique staff persons who attended at least one training, per program, was from 3 (21% of staff in that program) to 20 (77% of staff in that program). Leadership from all 4 intervention programs participated in the final learning community webinar.

For the 3 waitlist control programs, who received the intervention after the randomized trial, there were 21 attendees across 5 trainings (7 per program). This represented 15 unique staff persons (5 per program). Almost half of all eligible staff (46.8%, 15/32) attended at least 1 training. The range of unique staff persons who attended at least 1 training, per program, was from 2 (20% of staff in that program) to 8 (89% of staff in that program). Leadership from 2 of the 3 control programs participated in the final learning community webinar.

### Survey response rates

Across all programs, baseline, 6 months, and 12 months staff response rates were 79%, 76%, and 80%, respectively. Staff response rates by program ranged from 57 to 100%. Across all programs client response rates at baseline, 6 and 12 months were 91%, 97%, and 89%, respectively. Client response rates by program ranged from 64 to 100%. Staff and client characteristics at baseline, broken out by condition (Intervention, Control), are summarized in Supplemental Table 1.

### Participant characteristics at post-intervention

The primary comparison is between intervention and control conditions post-intervention, and Table 1 presents descriptive characteristics for staff (*N* = 74) and all clients (*N* = 174) at that timepoint. Rates of current smoking were 31.1% among staff and 70.7% among clients. For current smokers, mean CPD was 9.5 (SD = 4.7) among staff and 9.0 (SD = 6.0) among clients.

**Table 1:** Staff and client characteristics, post-intervention, in programs randomly assigned to intervention and control conditions^a^

	**Program staff** **(*n* = 74)**	**Program clients** **(*n* = 174)**
**Age**, mean (SD)	45.8 (10.7)	37.0 (11.1)
**Gender, %**		
Male	32 (43%) ^b^	129 (75%)
Female	42 (57%)	43 (25%)
**Race/ethnicity, %**		
Hispanic/Latino	18 (24%)	61 (35%)
Black or African American	16 (22%)	13 (7%)
White or Caucasian	35 (47%)	82 (47%)
Other/Multiple	5 (7%)	18 (10%)
**Education, %**		
Less than high school/GED	2 (3%)	44 (25%)
High school diploma or GED	45 (61%)	59 (34%)
More than high school/GED	27 (36%)	71 (41%)
**Smoking Status, %**		
Current Smokers	23 (31%)	123 (71%)
Former Smokers	39 (53%)	40 (23%)
Never Smokers	12 (16%)	11 (6%)
**Cigarettes per day**^c^	9.5 (4.7)	9.0 (6.0)
**Seriously thinking of quitting smoking?**^c^		
Yes, next 30 days	9 (39%)	42 (34%)
Yes, within the next 6 months	9 (39%)	37 (30%)
No, not thinking of quitting within the next 6 months	5 (22%)	43 (35%)

^a^ Comparison between conditions are conducted using post-intervention data only, and characteristics reported here refer to survey data collected at post-intervention only

^b^ Percentages rounded to nearest whole number

^c^ Includes current smokers only

Table 2 presents descriptive data for the outcome measures, for all programs combined, at the end of the randomized trial. Mean staff S-KAP scale values ranged from 2.77 (SD = 0.95) on the Practice scale to 3.61 (SD = 0.67) on the Belief scale. Most clients (79.2%) had been asked about smoking, while fewer had received tobacco-related counseling (48.1%) or medication (30.8%).

**Table 2:** Staff and client outcome measures, post-intervention, in programs randomly assigned to intervention and control conditions^a^

	**Program staff** **(*n* = 74)**	**Program clients** **(*n* = 174)**
	Mean (SD)	Mean (SD)
Staff smoking prevalence	23 (31%)	–
Staff belief scale	3.61 (0.67)	–
Staff self-efficacy scale	3.11 (0.55)	–
Staff practice scale	2.77 (0.95)	–
Client smoking prevalence	–	123 (71%)
Client was asked about smoking	–	137 (79%)
Client received tobacco-related counseling	–	63 (48%)
Client received cessation medication	–	41 (31%)

^a^ Comparison between conditions are conducted using post-intervention data only, and characteristics reported here refer to survey data collected at post-intervention only

### Comparison of outcomes by condition

Table 3 compares outcomes for programs assigned to intervention and control conditions. Staff smoking prevalence at post-intervention was 25% in intervention programs and 42.3% in control programs (*p* = 0.125). Intervention clients, as compared to controls, were less likely to report having received cessation medication (14.1% v. 60.4%) at 6 months.

**Table 3:** Comparison of outcomes for intervention and control programs post-intervention

	Intervention Group Four Programs	Control Group Three Programs	*χ*^*2*^*/t(df)*	*P* value
**Staff outcomes**	(*N* = 48)	(*N* = 26)		
Smoking prevalence	12 (25%) ^a^	11 (42%)	2.36 (1)	0.125
Staff belief scale	3.55 (0.67)	3.71 (0.68)	-1.00 (72)	0.323
Staff self-efficacy scale^b^	3.06 (0.56)	3.18 (0.53)	-0.73 (46)	0.472
Staff practice scale^b^	2.67 (0.98)	2.95 (0.90)	-0.95 (44)	0.345
**Client outcomes**	(*N* = 113)	(*N* = 61)		
Smoking Prevalence	80 (71%)	43 (70%)	0.00 (1)	0.966
Asked about smoking	87 (78%)	50 (82%)	0.44 (1)	0.507
Received tobacco-related counseling^c^	38 (46%)	25 (52%)	0.48 (1)	0.487
Received cessation medication^c^	12 (14%)	29 (60%)	30.8 (1)	< 0.0001

^a^ Percentages rounded to nearest whole number

^b^ Self-efficacy scale and Practice scale completed by clinical staff only

^c^ These items completed by current smokers and former smokers who quit in the treatment program

### Comparison of outcomes pre-post intervention collapsed across condition

Collapsing across conditions offers a larger sample with which to assess outcomes pre-post intervention. However, as there is no comparison group, any differences observed may not be attributed to the intervention.

Results (Table 4) show a decrease in staff smoking from 39.4% to 27% (OR = 0.50, 95% CI 0.31, 0.83, *p* = 0.008), and a decrease in client smoking from 76.8% to 67.1% (OR = 0.62, CI 0.41, 0.94, *p* = 0.025). Client-reported receipt of cessation medication decreased from 34.8% at pre- to 17.9% at post (OR = 0.49, 95% CI 0.31, 0.78, *p* = 0.003).

**Table 4:** Comparison of outcomes pre-post intervention for 7 programs, collapsed across condition^b^

	**Pre-intervention**	**Post-intervention**	**Post-intervention vs. Pre-intervention**^c^		
	**Mean (SD) or n(%)**		**OR (95%CI)**	***P* value**	**ICC or Phi**
**Staff outcomes**	(*N* = 71)	(*N* = 74)			
Smoking prevalence	28 (39%) ^a^	20 (27%)	0.50 (0.31, 0.83)	**0.008**	.26
			**Mean Difference (95%CI)**		
Staff belief scale	3.44 (0.72)	3.49 (0.70)	0.12 (-0.11, 0.34)	0.230	.36
Staff self-efficacy scale^d^	3.07 (0.63)	2.99 (0.58)	-0.04 (-0.28, 0.20)	0.277	.64
Staff practice scale^d^	2.51 (0.91)	2.58 (0.92)	0.16 (-0.27, 0.60)	0.454	.67
			**OR (95%CI)**		
**Client outcomes**	(*N* = 164)	(*N* = 149)			
Smoking Prevalence	126 (77%)	100 (67%)	0.62 (0.41, 0.94)	**0.025**	.22
Asked about smoking	118 (72%)	116 (78%)	1.50 (0.89, 2.53)	0.131	.29
Received any counseling^e^	64 (47%)	50 (45%)	0.93 (0.35, 2.45)	0.876	.30
Received any cessation medication^e^	47 (35%)	20 (18%)	0.49 (0.31, 0.78)	**0.003**	.60

^a^ Percentages rounded to nearest whole number

^b^ Adjusted for demographics (age, gender, race/ethnicity, and education); and also controlled for n esting of participants within clinics

^c^ Presented Odds Ratios for binary outcomes and Mean Differences for continuous outcomes

^d^ Clinical staff

^e^ Current smokers and former smokers who quit while in treatment

Given the small number of programs, large changes in a single program may influence overall findings. This is of interest because one control program (Program 5) implemented tobacco free grounds for reasons unrelated to the intervention. Sensitivity analyses repeated comparisons shown in Table 4, after removing data for Program 5. Results showed similar findings in reduced staff smoking (OR = 0.49, CI = 0.28, 0.88, *p* = 0.016) and reduced receipt of cessation medications among clients (OR = 0.41, CI = 0.19, 0.87, *p* = 0.019). However, the change in client smoking prevalence was no longer significant (OR = 0.61, CI = 0.34, 1.09, *p* = 0.098) and clients were more likely to report having been asked about their smoking at post (OR = 1.82, CI = 1.15, 2.86, *p* = 0.010).

## Discussion

Smoking prevalence in this sample, following the intervention, was 31.1% among staff and 70.7% among clients. This compares with a California smoking prevalence of 8.9% in 2020 [48]. For current smokers, mean CPD was 9.5 (SD = 4.7) among staff and 9.0 (SD = 6.0) among clients. When comparing intervention and control programs, we observed no difference by condition for 7 of 8 outcomes tested. The difference observed was that control clients more often reported receiving NRT in their treatment program. This may be explained by one control program (Program 5) required to implement tobacco free policies shortly after baseline, because it was operating on state-owned property. Of the 29 cases receiving cessation medication at 6 months, 24 were in Program 5. This means that the single difference observed between conditions is likely due to factors unrelated to the intervention.

Pre—post intervention comparisons, with programs collapsed across condition, showed decreased staff and client smoking prevalence, and decreased client receipt of cessation medication at post-intervention. However, sensitivity analyses eliminating Program 5 found only decreased staff smoking and decreased client receipt of cessation medication. A randomized trial design is scientifically more rigorous than a pre-post design. This leads us to the conservative conclusion that the multi-component intervention did not reduce staff or client smoking, and did not increase client receipt of tobacco-related services.

The reported pre-post intervention decrease in staff smoking may be associated with the COVID pandemic. Available data do not suggest a population-level decrease in smoking associated with the pandemic [49, 50]. However, residential SUD programs confronted significant disruption during the pandemic [51]. Some staff may have quit smoking, or smoking staff may have been less available to complete the post-intervention survey due to illness. There is also evidence that residential SUD clients received fewer clinical services early in the pandemic [51], consistent with fewer clients receiving cessation medication at post-intervention.

These findings may speak to what is required to implement or strengthen tobacco-related practices in SUD treatment. While the multi-component intervention did not show significant change in smoking prevalence, is it possible that the dose of the components was insufficient, or that these components would be effective if modified. For example, if incentives were required to be used for NRT, or if programs were required to incorporate further tobacco-related staff training or policy change. Other interventions, however, have demonstrated such change. Statewide implementation of tobacco free policies, as in New York and New Jersey, enlisted program licensing or certification requirements [16, 23]. In New York, mandated tobacco free policies were preceded by a lengthy planning period and supported by funding for staff training and NRT [23]. Guydish, Ziedonis et al. [24] found that a 6-month organizational change intervention was associated with increased tobacco-related services to clients, although client smoking prevalence was unchanged. An 18-month intervention including contractual requirements for tobacco-free grounds and provision of tobacco-cessation services, aided by financial support and consultation, was associated with decreased client smoking prevalence [22]. An intervention in Texas, which included staff training, no-cost NRT for programs, as well as support for strengthening tobacco policies, was associated with increased tobacco assessments reported by staff and increased use of NRT by clients [20, 52]. However, there is no research concerning the most effective combination of policies and practices needed to achieve these outcomes.

Findings may also speak to the importance of comparison conditions in studies of tobacco interventions in SUD treatment. Many such studies have used pre-post designs without comparison conditions [16, 23, 24, 52, 53], although some studies made use of naturalistic comparisons [22, 54]. Reliance on the pre-post analysis, in the current paper, would suggest an association between the intervention and lower staff smoking rates. However, this finding was not supported in clinical trial results.

Study limitations include limitations on statistical power. The cluster-randomized trial was designed to include 10 programs (5 intervention and 5 control programs). However, as given in Supplemental Figure 1, 12 programs were screened and only 7 were included, so that power to detect changes in the primary outcome of client smoking prevalence was suboptimal for the expected effect size. Further, as reflected in the sensitivity analyses, changes in one program may affect overall results. The utility of the randomized design was diminished by external factors driving one program to adopt tobacco-free policies independent of the study. Generalizability is restricted as all programs were California residential SUD programs, and all had expressed interest to better address smoking among program clients. Study procedures changed, due to COVID-19 pandemic restrictions, from on-site and online client survey procedures to use of paper surveys mailed to programs. While client response rates were high at all assessments, the direction of potential bias associated with this change is unknown. In this study 57% of eligible staff participated in at least one intervention session, and greater training exposure may achieve greater levels of change. However, residential programs must meet both operational and training requirements, and may be unable to release all staff to participate in scheduled trainings. Residential SUD programs experienced significant staffing challenges associated with the pandemic [51]. Performing COVID -related procedures such as assessment, testing, quarantine, and vaccination under conditions of diminished staffing likely reduced program abilities to address client smoking. Last, the intervention did not include specific strategies to increase use of NRT. Other studies suggest that increased access to NRT is accompanied by increased utilization among clients [17, 24], and findings of increased use of NRT occur in concert with decreased client smoking [22].

We conducted a cluster-randomized trial of a brief multi-component intervention to reduce smoking among staff and clients and increase client receipt of tobacco-related services. The intervention in this trial did not show significant impact on staff or client smoking behavior, or on tobacco-related services received by clients. To reduce smoking among staff and clients in SUD treatment, interventions should be supported by tobacco free grounds policies, regulatory or contractual requirements to provide tobacco services, programmatic funding to support those services, and/or access to NRT or other cessation medications.

## Supplementary Information

**Additional file 1: Supplemental Table 1.** Staff and client characteristics at baseline.**Supplemental Figure 1.** CONSORT Flow Diagram.

## Data Availability

Study data are available on request from the first author.
