# Supplementary material for: A cluster-randomized trial of a brief multi-component intervention to improve tobacco outcomes in substance use treatment
Source: Subst Abuse Treat Prev Policy. 2023 Jun 16;18:34. doi: 10.1186/s13011-023-00539-w (PMC10276468; doi:10.1186/s13011-023-00539-w)
Supplement: Supplementary file 1 — Additional file 1: Supplemental Table 1. Staff and client characteristics at baseline.Supplemental Figure 1. CONSORT Flow Diagram. [file 13011_2023_539_MOESM1_ESM.docx]

| Supplemental Table 1. Staff and client characteristics at baseline | | | | |
| --- | --- | --- | --- | --- |
|  | **Intervention Programs** | | **Control Programs** | |
|  | **Program staff**  **(n= 45)** | **Program clients**  **(n= 103)** | **Program staff**  **(n= 28)** | **Program clients**  **(n= 36)** |
| **Age**, mean (SD) | 47.5 (10.8) | 35.5 (10.6) | 40.5 (12.4) | 35.7 (8.7) |
| **Gender, %** |  |  |  |  |
| Male | 26 (58%)* | 88 (86%) | 4 (14%) | 13 (36%) |
| Female | 19 (42%) | 13 (13%) | 24 (86%) | 23 (64%) |
| Other | 0 (0%) | 1 (1%) | 0 (0.0%) |  |
| **Race/ethnicity, %** |  |  |  |  |
| Hispanic/Latino | 11 (25%) | 36 (35%) | 7 (26%) | 14 (39%) |
| Black or African American | 8 (18%) | 7 (7%) | 6 (22%) | 0 (0%) |
| White or Caucasian | 21 (48%) | 49 (48%) | 11 (41%) | 20 (56%) |
| Other/Multiple | 4 (9%) | 11 (11%) | 3 (11%) | 2 (6%) |
| **Education, %** |  |  |  |  |
| Less than high school/GED | 1 (2%) | 32 (31%) | 0 (0%) | 7 (19%) |
| High school diploma or GED | 31 (69%) | 41 (40%) | 11 (41%) | 11 (31%) |
| More than high school/GED | 13 (29%) | 30 (29%) | 16 (59%) | 18 (50%) |
|  |  |  |  |  |
| **Smoking Status, %** |  |  |  |  |
| Current Smokers | 17 (38%) | 83 (81%) | 5 (18%) | 22 (61.1%) |
| Former Smokers | 20 (44%) | 12 (12%) | 16 (57%) | 12 (33.3%) |
| Never Smokers | 8 (18%) | 8 (8%) | 7 (25%) | 2 (5.6%) |
| **Cigarettes per day**† | 8.4 (4.8) | 11.8 (9.1) | 7.4 (5.0) | 6.2 (4.8) |
| **Seriously thinking of quitting smoking?**† |  |  |  |  |
| Yes, next 30 days | 9 (53%) | 30 (36%) | 1 (20%) | 11 (50%) |
| Yes, within the next 6 months | 4 (23%) | 21 (25%) | 4 (80%) | 6 (27%) |
| No, not thinking of quitting within the next 6 months | 4 (23%) | 32 (39%) | 0 (0%) | 5 (23%) |
| * Percentages rounded to nearest whole number.  † Includes current smokers only | | | | |
